# Supplementary material for: Local audit of empiric antibiotic therapy in bacteremia: A retrospective cohort study
Source: PLoS One. 2021 Mar 18;16(3):e0248817. doi: 10.1371/journal.pone.0248817 (PMC7971877; doi:10.1371/journal.pone.0248817)
Supplement: S1 Table — (DOCX) [file pone.0248817.s002.docx]

**S1 Table. Susceptibility profile of *Pseudomonas aeruginosa* isolates**

|  | Ceftazidime | Piperacillin-Tazobactam | Meropenem | Ciprofloxacin | Tobramycin | Gentamicin |
| --- | --- | --- | --- | --- | --- | --- |
| 1 | Susceptible | Susceptible | Susceptible | Susceptible | Susceptible | Susceptible |
| 2 | Susceptible | Susceptible | Susceptible | Susceptible | Susceptible | Susceptible |
| 3 | Susceptible | Susceptible | Susceptible | Susceptible | Susceptible | Susceptible |
| 4 | Susceptible | Susceptible | Susceptible | Susceptible | Susceptible | Susceptible |
| 5 | Resistant | Susceptible | Resistant | Resistant | Susceptible | Susceptible |
| 6 | Susceptible | Susceptible | Resistant | Resistant | Susceptible | Intermediate |
| 7 | Susceptible | Susceptible | Susceptible | Susceptible | Susceptible | Susceptible |
| 8 | Susceptible | Susceptible | Susceptible | Susceptible | Susceptible | Susceptible |
| 9 | Susceptible | Susceptible | Susceptible | Susceptible | Susceptible | Susceptible |
| 10 | Susceptible | Susceptible | Susceptible | Susceptible | Susceptible | Susceptible |
| 11 | Susceptible | Susceptible | Intermediate | Resistant | Susceptible | Susceptible |
| 12 | Susceptible | Susceptible | Susceptible | Resistant | Susceptible | Susceptible |
