# Supplementary material for: Local audit of empiric antibiotic therapy in bacteremia: A retrospective cohort study
Source: PLoS One. 2021 Mar 18;16(3):e0248817. doi: 10.1371/journal.pone.0248817 (PMC7971877; doi:10.1371/journal.pone.0248817)
Supplement: S2 Table — (DOCX) [file pone.0248817.s003.docx]

**S2 Table. Description of unnecessary carbapenems**

| Case | Organism | Alternative and narrower antibiotic(s) other than carbapenem, to which the organism is susceptible to |
| --- | --- | --- |
| 1 | *Acinetobacter pittii* | Ceftazidime or Ciprofloxacin |
| 2 | *Stenotrophomonas* | Septra |
| 3 | *Alicaligenes faecalis, Aerococcus* | Piperacillin-Tazobactam |
| 4 | *Enterococcus faecalis* | Ampicillin or Piperacillin-Tazobactam |
| 5 | *Enterococcus faecalis* | Ampicillin or Piperacillin-Tazobactam |
| 6 | *Enterococcus faecalis* | Ampicillin or Piperacillin-Tazobactam |
| 7 | *Enterococcus faecalis* | Ampicillin or Piperacillin-Tazobactam |
| 8 | *Escherichia coli* | Ceftriaxone |
| 9 | *Escherichia coli* | Ceftriaxone |
| 10 | *Escherichia coli* | Ceftriaxone |
| 11 | *Escherichia coli* | Ceftriaxone |
| 12 | *Klebsiella pneumonia* | Ceftriaxone |
| 13 | *Klebsiella pneumonia* | Ceftriaxone |
| 14 | *Pseudomonas aeruginosa* | Piperacillin-Tazobactam |
| 15 | Methicillin-resistant *Staphylococcus aureus* | Vancomycin |
| 16 | Methicillin-resistant *Staphylococcus aureus* | Vancomycin |
| 17 | Methicillin-resistant *Staphylococcus aureus* | Vancomycin |
| 18 | *Proteus mirabilis* | Ceftriaxone |
| 19 | *Pseudomonas aeruginosa* | Ceftazidime |
| 20 | *Stenotrophomonas* | Septra |
| 21 | *Streptococcus pneumonia* | Ceftriaxone |
