# Supplementary material for: Local audit of empiric antibiotic therapy in bacteremia: A retrospective cohort study
Source: PLoS One. 2021 Mar 18;16(3):e0248817. doi: 10.1371/journal.pone.0248817 (PMC7971877; doi:10.1371/journal.pone.0248817)
Supplement: S3 Table — (DOCX) [file pone.0248817.s004.docx]

**S3 Table. Diagnostic accuracy of a combination of patient risk factors in predicting *Pseudomonas* bacteremia**

| Definition of high risk | Sensitivity | Specificity | PLR | NLR |
| --- | --- | --- | --- | --- |
| Hospital / healthcare associated or neutropenia | 0.92  (0.65-1.00) | 0.48  (0.41-0.55) | 1.77  (1.42-2.20) | 0.17  (0.03-1.14) |
| Hospital / healthcare associated or PICC line | 0.92  (0.65-1.00) | 0.47  (0.40-0.54) | 1.73  (1.39-2.15) | 0.18  (0.03-1.16) |
| Hospital / healthcare associated or *Pseudomonas* isolated from prior culture | 0.92  (0.65-1.00) | 0.49  (0.42-0.56) | 1.79  (1.43-2.23) | 0.17  (0.03-1.12) |
| Hospital / healthcare associated or *Pseudomonas* isolated from prior culture | 0.92  (0.65-1.00) | 0.41  (0.34-0.48) | 1.55  (1.26-1.90) | 0.21  (0.03-1.35) |
